# Supplementary material for: What are the barriers and facilitators to self-management of chronic conditions reported by women? A systematic review
Source: BMJ Open. 2022 Jul 20;12(7):e061655. doi: 10.1136/bmjopen-2022-061655 (PMC9305798; doi:10.1136/bmjopen-2022-061655)
Supplement: Supplementary data [file bmjopen-2022-061655supp002.pdf]

Supplementary material Two

Systematic Review Search Strategy

1. women.mp. or Women/
2. woman.mp. or Women/
3. Female/
4. 1 or 2 or 3
5. self-management.mp. or Self-Management/
6. 4 and 5
7. qualitative.mp.
8. 6 and 7
9. chronic.mp.
10. long-term.mp.
11. condition.mp.
12. illness.mp.
13. condition.mp.
14. Health/ or health.mp.
15. 9 or 10 or 11 or 12 or 13 or 14
16. 8 and 15
17. barrier*.mp.
18. facilitator*.mp.
19. 17 or 18
20. 16 and 19
21. limit 20 to english language
22. limit 21 to yr="2005 -Current"
