# Supplementary material for: What are the barriers and facilitators to self-management of chronic conditions reported by women? A systematic review
Source: BMJ Open. 2022 Jul 20;12(7):e061655. doi: 10.1136/bmjopen-2022-061655 (PMC9305798; doi:10.1136/bmjopen-2022-061655)
Supplement: Author's manuscript [file bmjopen-2022-061655.draft_revisions.pdf]

BMJ Open is committed to open peer review. As part of this commitment we make the peer review history of every article we publish publicly available.

When an article is published we post the peer reviewers' comments and the authors' responses online. We also post the versions of the paper that were used during peer review. These are the versions that the peer review comments apply to.

The versions of the paper that follow are the versions that were submitted during the peer review process. They are not the versions of record or the final published versions. They should not be cited or distributed as the published version of this manuscript.

BMJ Open is an open access journal and the full, final, typeset and author-corrected version of record of the manuscript is available on our site with no access controls, subscription charges or pay-per-view fees (http://bmjopen.bmj.com).

If you have any questions on BMJ Open's open peer review process please email info.bmjopen@bmj.com

BMJ Open

# What are the barriers and facilitators to self-management of chronic conditions reported by women? A systematic review.

| | |
|---|---|
| Journal: | *BMJ Open* |
| Manuscript ID | bmjopen-2022-061655 |
| Article Type: | Protocol |
| Date Submitted by the Author: | 31-Jan-2022 |
| Complete List of Authors: | Dwyer, Lucy; Manchester University NHS Foundation Trust, The Warrell Unit; The University of Manchester School of Health Sciences, Division of Nursing, Midwifery and Social Work<br>Dowding, Dawn; University of Manchester<br>Kearney, R; Manchester Academic Health Science Centre; University of Manchester |
| Keywords: | Urogynaecology < GYNAECOLOGY, Protocols & guidelines < HEALTH SERVICES ADMINISTRATION & MANAGEMENT, QUALITATIVE RESEARCH |
| | |

SCHOLARONE™
Manuscripts

# What are the barriers and facilitators to self-management of chronic conditions reported by women? A systematic review.

Dwyer. L[1,2], Dowding. D [2] and Kearney. R[1,3]

**Affiliations**:

[1] Saint Mary's Hospital, Manchester University NHS Foundation Trust, Manchester Academic Health Science Centre, Hathersage Road, Manchester M13 9WL, UK

[2]Division of Nursing, Midwifery and Social Work, School of Health Sciences, Faculty of Biology, Medicine and Health, University of Manchester, Oxford Road, Manchester, M13 9PL

[3] Institute of Human Development, Faculty of Medical & Human Sciences, University of Manchester, Oxford Road, Manchester, M13 9PL

Corresponding author: Lucy Dwyer

Lucy.dwyer@mft.nhs.uk 0161 276 6910

The Warrell Unit, Saint Mary's Hospital,

Manchester University NHS Foundation Trust

Hathersage Road

Manchester

M13 9WL

Dawn Dowding dawn.dowding@manchester.ac.uk

Rohna Kearney rohna.kearney@mft.nhs.uk

**Abstract word count:** 247

**Main article word Count:** 3087

**Key words:** Pessary, self-management, prolapse, systematic,

For peer review only

For peer review only

# What are the barriers and facilitators to self-management of chronic conditions reported by women? A systematic review.


## Abstract

### Introduction

Pelvic organ prolapse (POP) can be effectively managed using a pessary. A scoping review found that pessary self-management appears to benefit women with no increased risk. Despite this, many are unwilling to self-manage their pessary. At present there is a lack of understanding about what affects willingness to self-manage a pessary. However, there may be relevant, transferable findings from other literature about barriers to the self-management of other chronic conditions. Therefore, this systematic review aims to identify, appraise and synthesise the findings of published qualitative research exploring the barriers and facilitators to self-management of chronic conditions reported by women.

### Methods and analysis

The systematic review will be conducted and reported in accordance with PRISMA guidelines and a guide for the systematic review of qualitative data. A search of MEDLINE, CINAHL, EMBASE and PsycInfo will be undertaken to identify relevant articles which meet the eligibility criteria using the search terms 'Women', 'Woman' 'Female,' 'Chronic', 'Long-term', 'Disease', 'Illness', 'Condition' 'Health,' 'Self-management,' 'Qualitative,' 'Barrier' and 'Facilitator.' A hand search of the reference list of non-original research identified during the search but excluded, will be conducted for additional publications which meet the inclusion and exclusion criteria. Data relevant to the topic will be extracted and critical appraisal of all included publications undertaken.

### Ethics and dissemination

No ethical or Health Research Authority (HRA) approval is required to undertake the systematic review. The systematic review findings will be disseminated by publication. The findings will also inform subsequent exploratory work regarding pessary self-management.

For peer review only

Article Summary

Strengths and limitations of this study

- The review will be conducted using rigorous and reproducible methods to systematically identify, appraise and synthesise relevant qualitative evidence
- Critical appraisal of the evidence will be undertaken to determine the weighting of included research findings, as well as identifying the methodological strengths and limitations of the current evidence base
- The identification and synthesis of data will be limited to published articles found on the MEDLINE, CINAHL, EMBASE and PsycInfo databases and a hand search of reference lists. The authors will not seek original data
- The criteria that included studies must be published in English means there is potential for cultural bias in the findings

For peer review only

## Introduction

Pelvic organ prolapse (POP) is the downward displacement of one or more of the pelvic organs including the uterus, vaginal compartments, bowel or bladder [1]. Symptoms of POP include a vaginal bulge, heaviness or a dragging sensation, difficulties voiding or defecating and sexual dysfunction, all of which can significantly negatively impact a woman's quality of life [1]. A pessary is a medical device which can be inserted into the vagina to provide mechanical support to the prolapsed organs [2]. Pessaries offer women with prolapse a comparable improvement to surgery [3, 4]. However, the need for regular follow-up deters some women from this treatment option [5-7]. It has been suggested that women could be supported to self-manage their pessary, removing and inserting it independently, which would facilitate increased autonomy and potentially reduce the amount of face to face appointments required [8]. A scoping review conducted by the authors suggested pessary self-management appears to offer benefits such as comfort, convenience, increased perception of help and support and autonomy with no increased risk of complications (Dwyer et al, 2022 in press). However, the review also found that many women do not feel able, or lack willingness to self-manage their pessary, concluding that further research was required to explore barriers to self-management (Dwyer et al, 2022 in press).

<u>Rationale</u>

There is currently a lack of evidence about barriers to pessary self-management, however there may be relevant, transferable findings from other literature about barriers to the self-management of other chronic conditions. POP is a condition which solely affects women. Therefore, further understanding of the barriers to self-management specifically in women is indicated to ensure findings are relevant and generalisable to the female population. It is acknowledged that the intimate nature of pessary self-insertion and removal may contribute to condition specific barriers as identified in the scoping review (Dwyer et al, 2022 in press). However, it is also possible there are barriers to pessary self-management which apply to the concept of self-management of any chronic condition. The term self-management, was first used by Creer during the 1960s, to describe increased patient participation and engagement with care planning and treatment [9]. This definition of self-management has continued to evolve, and the current definition describes self-management as a procedure where a patient changes their behaviour through goal setting, information utilisation, decision making, action

For peer review only

and self-reaction with the aim of improving health, quality of life and increased self-efficacy [10]. The terms self-management and self-care have been used interchangeably and without clear definition, which has lead to confusion between both behaviours [9]. The World Health Organisation [11] and Department of Health [12], define self-care as actions to maintain and improve overall health as part of daily living [9]. Whereas self-management is condition focussed [9].

Objectives

This systematic review aims to identify, appraise and synthesise the findings of published qualitative research exploring the barriers and facilitators to self-management of chronic conditions reported by women. These findings will guide further research exploring, and intervention development to support women to overcome barriers to pessary self-management.

**Methods and analysis**

Qualitative research is the most appropriate evidence to explore the barriers and facilitators to self-management as it offers insight into the experiences and perceptions of individuals [13]. There are extensive tools and literature to guide the systematic review process of quantitative data, however there are fewer resources available to ensure a robust systematic review of qualitative data [14]. Therefore, in addition to using PRISMA guidance [15], a guide for the systematic review of qualitative data published by Butler, Hall [14] has also been used. This will ensure the systematic review process meets the high-quality standards established by PRISMA but also takes into account the methodological differences of qualitative and quantitative research.

In view of the focus upon qualitative publications, instead of the Participants, Intervention, Comparators and Outcomes (PICO) tool advocated by PRISMA [15], a modified tool will be used. Because qualitative studies do not have interventions and comparators, defining the relevant Population, Context and Outcome (PCO) is more appropriate [14]. Utilising the PCO tool, the population was specified as women aged 18 years or older, the context is studies exploring interventions designed to support the self-management of chronic conditions. The outcome is qualitative data reported by women which is relevant to barriers and facilitators

For peer review only

which influence the uptake, maintenance or concordance of the self-management intervention. Therefore, using the above parameters using the PCO tool the following research question was formulated: What are the barriers and facilitators to self-management of a chronic condition reported by women?

Registration

In accordance with PRISMA recommendations, this systematic review has been registered with The Open Science Framework (10.17605/OSF.IO/CTHSF) to ensure transparency and prevent contemporaneous duplication of the review [14, 15].

For peer review only

Information sources

The systematic qualitative review will be conducted in accordance with Preferred Reporting Items for Systematic Review and Meta-Analyses (PRISMA) guidelines [15]. A systematic search will be carried out using Medline, CINAHL, PsychINFO and Embase databases. These databases were identified following a discussion with an information technician at The University of Manchester library based upon their coverage of medical, nursing and allied health, psychology and biomedical journals.

**Figure One-Search Terms**

| **Population** | **Context-self-management** | **Context-chronic condition** | **Context-Qualitative methodology** | **Outcome** |
|---|---|---|---|---|
| Women | Self-management | Chronic | Qualitative | Barrier* |
| Woman | | Long-term | | Facilitator* |
| Female | | Disease | | |
| | | Illness | | |
| | | Condition | | |
| | | Health | | |

For peer review only

Eligibility criteria

Utilising the criteria detailed below, the reviewer will assess each identified publication's eligibility for inclusion. A PRISMA flow diagram will be used to present the screening process including the number of studies excluded and reasons for this to ensure transparency and reproducibility [15].

| Criteria | Explanation | Justification |
|---|---|---|
| Studies where participants are women | Studies with data from both men and women can be included if the findings are differentiated between men and women's responses. | POP is a condition which solely affects women. Therefore, to ensure the findings of this review can be generalised to the relevant population, it is necessary to limit included studies in case there are differences between the barriers and facilitators men and women report and experience to self-management interventions. |
| Studies where participants are aged 18 or over | Studies with data from individuals over and under 18 years can be included if the findings are differentiated between age groups. | The incidence of POP amongst women aged under 18 years is extremely low [16]. Therefore, the focus of research exploring pessary self-management will be women aged over 18 years. To ensure the findings of this review can be generalised to the relevant population, it is necessary to limit included studies in case there are differences between the barriers and facilitators adult women and young women and girls report and experience to self-management interventions. |

For peer review only - http://bmjopen.bmj.com/site/about/guidelines.xhtml

| | | |
|---|---|---|
| Studies exploring a self-management intervention | An intervention designed with the aim of improving health, quality of life or perceived self-efficacy for individuals with a specific condition through goal setting, information utilisation, decision making, action and self-reaction [10]. | The terms self-care and self-management have been poorly defined and used interchangeably [9] Therefore, it is necessary to ensure that self-management interventions included within the review meet Creer's definition [10] that a self-management intervention aims to improve health, quality of life or perceived self-efficacy for an individual with a specific health condition through either goal setting, information provision, supported decision making or self-reaction, to ensure the findings are valid. |
| Studies exploring a self-management intervention for a chronic condition | The term chronic disease has been poorly defined within both guidelines and literature leading to confusion [17]. Therefore, to ensure a standardised approach for the purpose of this review, a chronic (also known as a long-term) condition will be defined as a non-communicable condition that lasts for six months or longer and impacts upon quality of life [17-19]. | POP meets this definition of a chronic condition, therefore to ensure the findings of other studies included within the review are likely to be transferable to women with POP, it is necessary to exclude interventions for acute or terminal conditions as the barriers and facilitators cited may differ significantly to individuals with chronic conditions. |
| Studies which are qualitative research | Original qualitative research. Qualitative studies, mixed methods studies and data collected by questionnaire can be included if answered in free text and analysed using a qualitative approach. | Qualitative research is the most appropriate evidence to explore the barriers and facilitators to self-management as it offers insight into the experiences and perceptions of individuals [13]. |

For peer review only

| | | |
|---|---|---|
| Studies which have findings related to the barriers or facilitators to a self-management intervention | The results will include findings about the barriers and facilitators which influence uptake and/or maintenance and /or concordance with a self-management intervention. | The aim of the review is to identify the barriers and facilitators to self-management interventions. |
| Studies with finding directly reported by the target population of women | Data about barriers and facilitators to a self-management intervention must be collected directly from the study population rather than healthcare professionals, partners, family members or carers. | This systematic review aims to identify barriers and facilitators to self-management interventions from the perspective of the women they are designed for. Therefore, the perspectives of healthcare professionals, partners, family members or carers will not be included as these interpretations may not accurately reflect the experiences of women. |
| Language | Studies must be published in the English language | For pragmatic reasons, studies published in languages other than English will be excluded from the review due to the cost and time required for translation. |

For peer review only

| | | |
|---|---|---|
| Original research | Publications that are not original research including reviews, case reports and commentary articles will not be included. | Non-original research will be excluded to avoid duplication of results and ensure all included publications provide rigorous evidence rather than subjective opinion. |
| Date range | Eligible studies will be published between 2005 and the date of the search. | Since 2005, United Kingdom (UK) key health policy demonstrates a distinct change from a traditional medical model approach to healthcare to a person-centred approach where individuals are empowered to self-care and self-manage their long-term conditions with the goal of providing a more efficient service [12, 20, 21]. The NHS Long-term plan, launched in 2019, refers to supported self-management as a means to meet healthcare delivery goals and improve patient care [22], demonstrating that self-management remains a key aspect of national healthcare policy. Therefore, the time parameters for the search will be from 2005 until the search date. It is anticipated this will ensure review findings are sufficiently current to reflect recent practice and experience of women. |

For peer review only

Data management and selection

Following the search, all identified abstracts will be uploaded to reference manager software, following which duplicates and non-original research publications will be removed. The abstracts will then be reviewed for relevance to the review question and in accordance with the eligibility criteria. A sample of 20% of abstracts will be screened by an independent reviewer to ensure concordance with decisions about eligibility. In the instance of disagreement regarding included or excluded studies not resolved through discussion, a third reviewer will be asked to make the final decision.  Potentially relevant sources will be retrieved in full and assessed in detail against the inclusion criteria. Reasons for exclusion of sources of evidence that do not meet the inclusion criteria will be recorded and reported in the systematic review. The reference list of all review articles identified in the search and excluded due to not being original research, will be checked and all relevant original research cited will be reviewed for potential inclusion within this review.

Data collection

Data will be extracted using a data extraction tool developed by the reviewers based upon qualitative systematic review guidance [14, 23]. The extracted data will be entered into the tool electronically via Microsoft Excel. The data extraction form will include details of the author(s), year of publication, methodology utilised, theoretical orientation of the researchers, population studied, , the self-management intervention, the chronic condition targeted, and key findings relevant to the barriers and facilitators of the self-management intervention. Study findings will be recorded in two separate columns to differentiate between first and second order constructs maintaining clarity about what findings are raw data and which are researcher interpretations [14].

A second reviewer will perform data extraction from a subset of 10% of included articles to ensure a standardised, reproducible approach. In the instance of discrepancies in data extraction processes, these will be explored, discussed and changes made to the data extraction tool if necessary. Any amendments to the data extraction tool will be recorded and published with the review findings to ensure transparency. The final version of the data extraction tool including extracted data will be published alongside the review findings to ensure reproducibility.

For peer review only

Data synthesis

The extracted data will be analysed using a thematic approach. Findings will be coded line by line to identify emerging and recurring descriptive themes amongst the studies [24]. Once all extracted data has been coded, the descriptive themes identified from the coding will be further analysed for broader analytic themes that span the included studies [24]. A number of qualitative systematic reviews of barriers and facilitators to healthy behaviour have used thematic synthesis [25]. Therefore, the synthesis will be performed utilising this established process to ensure an established inductive and deductive approach [25].

Quality appraisal

Qualitative findings offers insight into individuals' views, experiences and perspectives and therefore do not attempt to be generalisable or replicable [26]. However, rigour can achieved in qualitative research by ensuring findings are trustworthy, authentic, typical, transferable and valid [26]. Butler, Hall [14] describe their use of the Critical Appraisal Skills Programme (CASP) qualitative checklist [27]. CASP specify they have not suggested a scoring system for appraising qualitative studies as this was not the purpose of the checklist [27]. However, Butler, Hall [14] explain the scoring system they successfully used for each checklist criterion and overall scoring to determine the level of quality of each individual study. Therefore, this same scoring system of the CASP checklist will be utilised (Figure Two). As this review aims to provide an overview of the current evidence base, all studies will be included regardless of the appraised quality [14]. However, as advocated by Long, French [28] a hierarchical approach to thematic analysis will be undertaken with the quality of a study informing whether or not new codes are created. For this review, new codes will be created for high and medium quality studies. However, for low and very low-quality studies, no new codes will be created but findings utilised to support themes identified from codes created through thematic analysis of higher quality studies. This will ensure finding are weighted appropriately based upon the methodological quality of the studies [28]. Furthermore, an overview of strengths and limitations of the identified evidence base will be provided to highlight any patterns in quality, or lack of it, and inform the methodological design of future research within this subject area.

For peer review only

**Figure Two-Quality Appraisal Scoring System**

| **CASP Scoring system** | |
|---|---|
| Checklist criterion | |
| Yes | 1 |
| No | 0 |
| Unsure | 0.5 |
| Overall score | |
| High quality | 9-10 |
| Moderate quality | 7.5-8.5 |
| Low quality | 6- ≤7.5 |
| Very low quality | ≤6 |

For peer review only

Another issue with the synthesis of findings from qualitative studies is that the subjectivity of study findings means our confidence in cumulative evidence synthesised from included studies is reliant upon decisions and interpretations made by authors. While quality appraisal using the CASP tool assesses research processes, it does not determine the credibility or dependability of interpretations made by the researcher which may be influenced by personal bias [29]. Because of this, researchers from the Joanna Briggs Institute developed a system called ConQual which enables assessment and scoring of a study's credibility, dependability and therefore overall confidence in the synthesised findings [29]. Using the ConQual, statements made from the findings of synthesised data will be presented in tabular format alongside a ConQual rating of credibility, dependability and overall score to ensure conclusions made can be weighted accordingly.

Outcomes and prioritisation

The experiences and perceptions of women reporting barriers and facilitators to self-management interventions targeting chronic diseases are the focus of this review. Therefore, all, barriers or facilitators to the uptake or maintenance of a self-management intervention for a chronic condition reported within identified and eligible studies will be included within data synthesis.

Data Analysis and Presentation

Details of the studies identified by the search, for example the extent of identified literature, the context of included research such as the methodology utilised, philosophical standpoint, country of origin, study population, self-management intervention and chronic disease targeted, will be presented in numerical and tabular format to provide an overview of the evidence base. A CASP and ConQual ratings will also be presented alongside each included study to enable readers to take in to account the quality, dependability, credibility and overall confidence in each study and therefore synthesised findings. Themes identified will be described in both tabular and text format to ensure transparency and clarity in the process of thematic analysis and identified themes, in addition to more detailed discussion and demonstration of evidence supporting identified themes within the text.

Confidence in cumulative evidence

One criticism of the synthesis of qualitative studies is that the nature of qualitative research means the context of each study may vary significantly, impacting upon study findings which creates a potential for bias when performing meta-synthesis [24]. To minimise this, the extraction tool will include details of each study’s context and be presented alongside the review findings, enabling readers to contextualise study findings as advocated by Thomas and Harden [24]. Further sub-analysis will be undertaken to explore whether certain contexts, for example the age of a study population, type of self-management intervention or chronic condition correlate with specific themes. This will further understanding about whether identified barriers or facilitators relate to a specific context or have broader applicability [24].

Patient and public involvement

Members of the public have not directly been involved with development of this protocol or review process. However, the need for research exploring pessary self-management was highlighted by The James Lind Alliance (JLA) Priority Setting Partnership for pessary and prolapse [30]. Several women with experience of pessaries participated in this partnership either as members of the steering group, by attending the consensus workshop or completing questionnaires. Understanding more about pessary self-management was

For peer review only - http://bmjopen.bmj.com/site/about/guidelines.xhtml

ranked third out of 20 priorities by the JLA Priority Setting Partnership. This systematic review will inform a research project exploring pessary self-management which has been identified and prioritised by patients and members of the public.

Ethics and dissemination

No ethical or Health Research Authority (HRA) approval is required to undertake the systematic review. The systematic review findings will be disseminated by publication in a peer reviewed journal. The findings will also inform subsequent exploratory work regarding pessary self-management.

For peer review only

## Authors contributions

Lucy Dwyer devised the systematic review question, methodology and drafted this manuscript.

Dawn Dowding substantively contributed to the development of the systematic review question, methodology and revised and approved this manuscript.

Rohna Kearney substantively contributed to the development of the systematic review question, methodology and revised and approved this manuscript.

## Competing interests statement

Lucy Dwyer and Rohna Kearney are co-applicants of the NIHR/HTA funded Treatment of Prolapse with Self-Care Pessary (TOPSY) study.

## Funding

Lucy Dwyer, Clinical Doctoral Research Fellow, NIHR300519 is funded by Health Education England (HEE) / National Institute for Health Research (NIHR) for this research project. The views expressed in this publication are those of the author(s) and not necessarily those of the NIHR, NHS or the UK Department of Health and Social Care.

For peer review only

## References


1. Haylen, B.T., et al., *An International Urogynecological Association (IUGA) / International Continence Society (ICS) Joint Report on the Terminology for Female Pelvic Organ Prolapse (POP)*, in *Neurourology and Urodynamics*. 2016.
2. Bugge, C., et al., *Pessaries (mechanical devices) for managing pelvic organ prolapse in women.* Cochrane Database Syst Rev, 2020. **11**(11): p. Cd004010.
3. Lone, F., R. Thakar, and A.H. Sultan, *One-year prospective comparison of vaginal pessaries and surgery for pelvic organ prolapse using the validated ICIQ-VS and ICIQ-UI (SF) questionnaires.* International Urogynecology Journal, 2015. **26**(9).
4. Miceli, A. and J.L. Dueñas-Diez, *Effectiveness of ring pessaries versus vaginal hysterectomy for advanced pelvic organ prolapse. A cohort study.* International Urogynecology Journal, 2019. **30**(12).
5. Kearney, R. and C. Brown, *Self-management of vaginal pessaries for pelvic organ prolapse.* BMJ Quality Improvement Reports, 2014. **3**(1).
6. Basu, M., B. Wise, and J. Duckett, *A qualitative study of women's preferences for treatment of pelvic floor disorders.* BJOG: An International Journal of Obstetrics & Gynaecology, 2011. **118**(3): p. 338-344.
7. Nguyen, J.N. and C.R. Jones, *Pessary treatment of pelvic relaxation: Factors affecting successful fitting and continued use.* Journal of Wound, Ostomy and Continence Nursing, 2005. **32**(4).
8. Bugge, C., et al., *Does self-management of vaginal pessaries improve care for women with pelvic organ prolapse?*, in *The BMJ*. 2021.
9. Rijken, M., et al., *Supporting self-management.* Caring for people with chronic conditions: a health system perspective, 2008: p. 116-142.
10. Creer, T.L., *Self-management of chronic illness*, in *Handbook of self-regulation*. 2000, Elsevier. p. 601-629.
11. Organization, W.H., *Health education in self-care: Possibilities and limitations*. 1984, World Health Organization.
12. Department of Health, *Self Care: A Real Choice*, London: Department of Health Publications, Editor. 2005.
13. Gray, J. and S. Grove, *Burns and Grove's the practice of nursing research : appraisal, synthesis, and generation of evidence*. 2021.
14. Butler, A., H. Hall, and B. Copnell, *A Guide to Writing a Qualitative Systematic Review Protocol to Enhance Evidence-Based Practice in Nursing and Health Care.* Worldviews Evid Based Nurs, 2016. **13**(3): p. 241-9.
15. Moher, D., et al., *Preferred reporting items for systematic review and meta-analysis protocols (PRISMA-P) 2015 statement.* Systematic Reviews, 2015. **4**(1): p. 1.
16. Hines, M., R. High, and J.M. Danford, *Management of Pelvic Floor Disorders in Young Women.* Current Bladder Dysfunction Reports, 2020. **15**(4): p. 259-266.
17. Bernell, S. and S.W. Howard, *Use Your Words Carefully: What Is a Chronic Disease?* Frontiers in public health, 2016. **4**: p. 159-159.
18. Department of Health, *Long Term Conditions Compendium of Information*. 2012.
19. O'Halloran, J., G.C. Miller, and H. Britt, *Defining chronic conditions for primary care with ICPC-2.* Family Practice, 2004. **21**(4): p. 381-386.
20. Department of Health, *Supporting people with long term conditions to self care – A guide to developing local strategies and good practice*, T.D.o. Health, Editor. 2006.
21. Department of Health, *The NHS Plan: a plan for investment a plan for reform*, D.o. Health, Editor. 2007.
22. Nhs, *The NHS Long Term Plan*. 2019.
23. Timulak, L., *Meta-analysis of qualitative studies: A tool for reviewing qualitative research findings in psychotherapy.* Psychotherapy Research, 2009. **19**(4-5): p. 591-600.


For peer review only

24. Thomas, J. and A. Harden, *Methods for the thematic synthesis of qualitative research in systematic reviews.* BMC Medical Research Methodology, 2008. **8**(1): p. 45.
25. Kanavaki, A.M., et al., *Barriers and facilitators to physical activity in people with hip or knee osteoarthritis: protocol for a systematic review of qualitative evidence.* BMJ Open, 2016. **6**(11): p. e012049.
26. Holloway, I. and K. Galvin, *Qualitative research in nursing and healthcare*. 2016: John Wiley & Sons.
27. Critical Appraisal Skills Programme (CASP). *Qualitative checklist*. 2018 08/01/22]; Available from: https://casp-uk.net/wp-content/uploads/2018/01/CASP-Qualitative-Checklist-2018.pdf.
28. Long, H.A., D.P. French, and J.M. Brooks, *Optimising the value of the critical appraisal skills programme (CASP) tool for quality appraisal in qualitative evidence synthesis.* Research Methods in Medicine & Health Sciences, 2020. **1**(1): p. 31-42.
29. Munn, Z., et al., *Establishing confidence in the output of qualitative research synthesis: the ConQual approach.* BMC Medical Research Methodology, 2014. **14**(1): p. 108.
30. Lough, K., et al., *Shared research priorities for pessary use in women with prolapse: Results from a James Lind Alliance Priority Setting Partnership.* BMJ Open, 2018. **8**(4).

For peer review only

# PRISMA 2020 Checklist

| Section and Topic | Item # | Checklist item | Location where item is reported |
|---|---|---|---|
| **TITLE** | | | |
| Title | 1 | Identify the report as a systematic review. | Page 1 |
| **ABSTRACT** | | | |
| Abstract | 2 | See the PRISMA 2020 for Abstracts checklist. | Page 2 |
| **INTRODUCTION** | | | |
| Rationale | 3 | Describe the rationale for the review in the context of existing knowledge. | Page 4 |
| Objectives | 4 | Provide an explicit statement of the objective(s) or question(s) the review addresses. | Page 5 |
| **METHODS** | | | |
| Eligibility criteria | 5 | Specify the inclusion and exclusion criteria for the review and how studies were grouped for the syntheses. | Page 8 |
| Information sources | 6 | Specify all databases, registers, websites, organisations, reference lists and other sources searched or consulted to identify studies. Specify the date when each source was last searched or consulted. | Page 7 |
| Search strategy | 7 | Present the full search strategies for all databases, registers and websites, including any filters and limits used. | To be included with results |
| Selection process | 8 | Specify the methods used to decide whether a study met the inclusion criteria of the review, including how many reviewers screened each record and each report retrieved, whether they worked independently, and if applicable, details of automation tools used in the process. | Page 12 |
| Data collection process | 9 | Specify the methods used to collect data from reports, including how many reviewers collected data from each report, whether they worked independently, any processes for obtaining or confirming data from study investigators, and if applicable, details of automation tools used in the process. | Page 12 |
| Data items | 10a | List and define all outcomes for which data were sought. Specify whether all results that were compatible with each outcome domain in each study were sought (e.g. for all measures, time points, analyses), and if not, the methods used to decide which results to collect. | Page 12 |
| | 10b | List and define all other variables for which data were sought (e.g. participant and intervention characteristics, funding sources). Describe any assumptions made about any missing or unclear information. | Page 12 |
| Study risk of bias assessment | 11 | Specify the methods used to assess risk of bias in the included studies, including details of the tool(s) used, how many reviewers assessed each study and whether they worked independently, and if applicable, details of automation tools used in the process. | Page 13-14 |
| Effect measures | 12 | Specify for each outcome the effect measure(s) (e.g. risk ratio, mean difference) used in the synthesis or presentation of results. | Page 13-14 |
| Synthesis methods | 13a | Describe the processes used to decide which studies were eligible for each synthesis (e.g. tabulating the study intervention characteristics and comparing against the planned groups for each synthesis (item #5)). | Page 15 |
| | 13b | Describe any methods required to prepare the data for presentation or synthesis, such as handling of missing summary statistics, or data conversions. | Page 15 |
| | 13c | Describe any methods used to tabulate or visually display results of individual studies and s 4yntheses. | Page 15 |
| | 13d | Describe any methods used to synthesize results and provide a rationale for the choice(s). If meta-analysis was performed, describe the model(s), method(s) to identify the presence and extent of statistical heterogeneity, and software package(s) used. | Page 14-15 |
| | 13e | Describe any methods used to explore possible causes of heterogeneity among study results (e.g. subgroup analysis, meta-regression). | Page 15 |
| | 13f | Describe any sensitivity analyses conducted to assess robustness of the synthesized results. | Page 15 |
| Reporting bias assessment | 14 | Describe any methods used to assess risk of bias due to missing results in a synthesis (arising from reporting biases). | Page 15 |

For peer review only - http://bmjopen.bmj.com/site/about/guidelines.xhtml

# PRISMA 2020 Checklist

| Section and Topic | Item # | Checklist item | Location where item is reported |
|---|---|---|---|
| Certainty assessment | 15 | Describe any methods used to assess certainty (or confidence) in the body of evidence for an outcome. | Page 15 |
| **RESULTS** | | | |
| Study selection | 16a | Describe the results of the search and selection process, from the number of records identified in the search to the number of studies included in the review, ideally using a flow diagram. | To be included with results |
| | 16b | Cite studies that might appear to meet the inclusion criteria, but which were excluded, and explain why they were excluded. | To be included with results |
| Study characteristics | 17 | Cite each included study and present its characteristics. | To be included with results |
| Risk of bias in studies | 18 | Present assessments of risk of bias for each included study. | To be included with results |
| Results of individual studies | 19 | For all outcomes, present, for each study: (a) summary statistics for each group (where appropriate) and (b) an effect estimate and its precision (e.g. confidence/credible interval), ideally using structured tables or plots. | To be included with results |
| Results of syntheses | 20a | For each synthesis, briefly summarise the characteristics and risk of bias among contributing studies. | To be included with results |
| | 20b | Present results of all statistical syntheses conducted. If meta-analysis was done, present for each the summary estimate and its precision (e.g. confidence/credible interval) and measures of statistical heterogeneity. If comparing groups, describe the direction of the effect. | To be included with results |
| | 20c | Present results of all investigations of possible causes of heterogeneity among study results. | To be included with results |
| | 20d | Present results of all sensitivity analyses conducted to assess the robustness of the synthesized results. | To be included with results |
| Reporting biases | 21 | Present assessments of risk of bias due to missing results (arising from reporting biases) for each synthesis assessed. | To be included with results |
| Certainty of evidence | 22 | Present assessments of certainty (or confidence) in the body of evidence for each outcome assessed. | To be included with results |
| **DISCUSSION** | | | |
| Discussion | 23a | Provide a general interpretation of the results in the context of other evidence. | To be included with results |

For peer review only

# PRISMA 2020 Checklist

| Section and Topic | Item # | Checklist item | Location where item is reported |
|---|---|---|---|
| | 23b | Discuss any limitations of the evidence included in the review. | To be included with results |
| | 23c | Discuss any limitations of the review processes used. | To be included with results |
| | 23d | Discuss implications of the results for practice, policy, and future research. | To be included with results |
| **OTHER INFORMATION** | | | |
| Registration and protocol | 24a | Provide registration information for the review, including register name and registration number, or state that the review was not registered. | Page 6 |
| | 24b | Indicate where the review protocol can be accessed, or state that a protocol was not prepared. | This publication |
| | 24c | Describe and explain any amendments to information provided at registration or in the protocol. | n/a |
| Support | 25 | Describe sources of financial or non-financial support for the review, and the role of the funders or sponsors in the review. | Page 17 |
| Competing interests | 26 | Declare any competing interests of review authors. | Page 17 |
| Availability of data, code and other materials | 27 | Report which of the following are publicly available and where they can be found: template data collection forms; data extracted from included studies; data used for all analyses; analytic code; any other materials used in the review. | To be included with results |

*From:* Page MJ, McKenzie JE, Bossuyt PM, Boutron I, Hoffmann TC, Mulrow CD, et al. The PRISMA 2020 statement: an updated guideline for reporting systematic reviews. BMJ 2021;372:n71. doi: 10.1136/bmj.n71

For more information, visit: http://www.prisma-statement.org/

For peer review only

# What are the barriers and facilitators to self-management of chronic conditions reported by women? A systematic review.

| | |
|---|---|
| Journal: | *BMJ Open* |
| Manuscript ID | bmjopen-2022-061655.R1 |
| Article Type: | Protocol |
| Date Submitted by the Author: | 29-Apr-2022 |
| Complete List of Authors: | Dwyer, Lucy; Manchester University NHS Foundation Trust, The Warrell Unit; The University of Manchester School of Health Sciences, Division of Nursing, Midwifery and Social Work<br>Dowding, Dawn; University of Manchester<br>Kearney, R; Manchester Academic Health Science Centre; University of Manchester |
| <b>Primary Subject Heading</b>: | Obstetrics and gynaecology |
| Secondary Subject Heading: | Nursing |
| Keywords: | Urogynaecology < GYNAECOLOGY, Protocols & guidelines < HEALTH SERVICES ADMINISTRATION & MANAGEMENT, QUALITATIVE RESEARCH |
| | |

SCHOLARONE™
Manuscripts

# What are the barriers and facilitators to self-management of chronic conditions reported by women? A systematic review.

Dwyer. L[1,2], Dowding. D [2] and Kearney. R[1,3]

**Affiliations**:

[1] Saint Mary's Hospital, Manchester University NHS Foundation Trust, Manchester Academic Health Science Centre, Hathersage Road, Manchester M13 9WL, UK

[2]Division of Nursing, Midwifery and Social Work, School of Health Sciences, Faculty of Biology, Medicine and Health, University of Manchester, Oxford Road, Manchester, M13 9PL

[3] Institute of Human Development, Faculty of Medical & Human Sciences, University of Manchester, Oxford Road, Manchester, M13 9PL

Corresponding author: Lucy Dwyer

Lucy.dwyer@mft.nhs.uk 0161 276 6910

The Warrell Unit, Saint Mary's Hospital,

Manchester University NHS Foundation Trust

Hathersage Road

Manchester

M13 9WL

Dawn Dowding dawn.dowding@manchester.ac.uk

Rohna Kearney rohna.kearney@mft.nhs.uk

**Abstract word count:** 281

**Main article word Count:** 3096

**Key words:** Pessary, self-management, prolapse, systematic,

For peer review only

For peer review only

# What are the barriers and facilitators to self-management of chronic conditions reported by women? A systematic review.


## Abstract

Introduction

Pelvic organ prolapse (POP) can be effectively managed using a pessary. A scoping review found that pessary self-management appears to benefit women with no increased risk. Despite this, many are unwilling to self-manage their pessary. At present there is a lack of understanding about what affects willingness to self-manage a pessary. However, there may be relevant, transferable findings from other literature about barriers to the self-management of other chronic conditions. Therefore, this systematic review aims to identify, appraise and synthesise the findings of published qualitative research exploring the barriers and facilitators to self-management of chronic conditions reported by women.

Methods and analysis

The systematic review will be conducted and reported in accordance with PRISMA guidelines and a guide for the systematic review of qualitative data. A search of MEDLINE, CINAHL, EMBASE and PsycInfo will be undertaken to identify relevant articles which meet the eligibility criteria using the search terms ‘Women’, ‘Woman’ ‘Female,’ ‘Chronic’, ‘Long-term’, ‘Disease’, ‘Illness’, ‘Condition’ ‘Health,’ ‘Self-management,’ ‘Qualitative,’ ‘Barrier’ and ‘Facilitator.’ A hand search of the reference list of non-original research identified during the search but excluded, will be conducted for additional publications which meet the inclusion and exclusion criteria. Studies published before 2005 and those not available in English will be excluded. Data relevant to the topic will be extracted and critical appraisal of all included publications undertaken.

Ethics and dissemination


For peer review only

No ethical or Health Research Authority (HRA) approval is required to undertake the systematic review. The systematic review findings will be disseminated by publication. The findings will also inform subsequent exploratory work regarding pessary self-management.

Registration

In accordance with PRISMA recommendations, this systematic review has been registered with The Open Science Framework (10.17605/OSF.IO/CTHSF) and PROSPERO (CRD42022327643).

For peer review only

Article Summary

Strengths and limitations of this study

- The review will be conducted using rigorous and reproducible methods to systematically identify, appraise and synthesise relevant qualitative evidence
- Critical appraisal of the evidence will be undertaken to determine the weighting of included research findings, as well as identifying the methodological strengths and limitations of the current evidence base
- The identification and synthesis of data will be limited to published articles found on the MEDLINE, CINAHL, EMBASE and PsycInfo databases and a hand search of reference lists. The authors will not seek original data
- The criteria that included studies must be published in English means there is potential for cultural bias in the findings

For peer review only

## Introduction

Pelvic organ prolapse (POP) is the downward displacement of one or more of the pelvic organs including the uterus, vaginal compartments, bowel or bladder [1]. Symptoms of POP include a vaginal bulge, heaviness or a dragging sensation, difficulties voiding or defecating and sexual dysfunction, all of which can significantly negatively impact a woman's quality of life [1]. A pessary is a medical device which can be inserted into the vagina to provide mechanical support to the prolapsed organs [2]. Pessaries offer women with prolapse a comparable improvement to surgery [3, 4]. However, the need for regular follow-up deters some women from this treatment option [5-7]. It has been suggested that women could be supported to self-manage their pessary, removing and inserting it independently, which would facilitate increased autonomy and potentially reduce the amount of face to face appointments required [8]. A scoping review conducted by the authors suggested pessary self-management appears to offer benefits such as comfort, convenience, increased perception of help and support and autonomy with no increased risk of complications (Dwyer et al, 2022 in press). However, the review also found that many women do not feel able, or lack willingness to self-manage their pessary, concluding that further research was required to explore barriers to self-management (Dwyer et al, 2022 in press).

For peer review only

### Rationale

There is currently a lack of evidence about barriers to pessary self-management, however there may be relevant, transferable findings from other literature about barriers to the self-management of other chronic conditions. POP is a condition which solely affects women. Therefore, further understanding of the barriers to self-management specifically in women is indicated to ensure findings are relevant and generalisable to the female population. It is acknowledged that the intimate nature of pessary self-insertion and removal may contribute to condition specific barriers as identified in the scoping review (Dwyer et al, 2022 in press). However, it is also possible there are barriers to pessary self-management which apply to the concept of self-management of any chronic condition. The term self-management, was first used by Creer during the 1960s, to describe increased patient participation and engagement with care planning and treatment [9]. This definition of self-management has continued to evolve, and the current definition describes self-management as a procedure where a patient changes their behaviour through goal setting, information utilisation, decision making, action

and self-reaction with the aim of improving health, quality of life and increased self-efficacy [10]. The terms self-management and self-care have been used interchangeably and without clear definition, which has lead to confusion between both behaviours [9]. The World Health Organisation [11] and United Kingdom Department of Health [12], define self-care as actions to maintain and improve overall health as part of daily living [9]. Whereas self-management is condition focussed [9].

Objectives

This systematic review aims to identify, appraise and synthesise the findings of published qualitative research exploring the barriers and facilitators to self-management of chronic conditions reported by women. These findings will guide further research exploring, and intervention development to support women to overcome barriers to pessary self-management.

## Methods and analysis

Qualitative research is the most appropriate evidence to explore the barriers and facilitators to self-management as it offers insight into the experiences and perceptions of individuals [13]. There are extensive tools and literature to guide the systematic review process of quantitative data, however there are fewer resources available to ensure a robust systematic review of qualitative data [14]. Therefore, in addition to using PRISMA guidance (supplemental file 1) [15], a guide for the systematic review of qualitative data has also been used[14]. This will ensure the systematic review process meets the high-quality standards established by PRISMA but also takes into account the methodological differences of qualitative and quantitative research.

In view of the focus upon qualitative publications, instead of the Participants, Intervention, Comparators and Outcomes (PICO) tool advocated by PRISMA [15], a modified tool will be used. Because qualitative studies do not have interventions and comparators, defining the relevant Population, Context and Outcome (PCO) is more appropriate [14]. Utilising the PCO tool, the population was specified as women aged 18 years or older, the context is studies exploring interventions designed to support the self-management of chronic conditions. The outcome is qualitative data reported by women which is relevant to barriers and facilitators

For peer review only

which influence the uptake, maintenance or concordance of the self-management intervention. Therefore, using the above parameters using the PCO tool the following research question was formulated: What are the barriers and facilitators to self-management of a chronic condition reported by women?

Registration

In accordance with PRISMA recommendations, this systematic review has been registered with The Open Science Framework (10.17605/OSF.IO/CTHSF) and PROSPERO (CRD42022327643) to ensure transparency and prevent contemporaneous duplication of the review [14, 15].

For peer review only

Information sources

The systematic qualitative review will be conducted in accordance with Preferred Reporting Items for Systematic Review and Meta-Analyses (PRISMA) guidelines [15] (supplemental file 1). A systematic search of Medline, CINAHL, PsychINFO and Embase databases will be carried out using the search terms detailed in table one (supplemental file 2). These databases were identified following a discussion with an information technician at The University of Manchester library based upon their coverage of medical, nursing and allied health, psychology and biomedical journals.

**Table One-Search Terms**

| Population | Context-self-management | Context-chronic condition | Context-Qualitative methodology | Outcome |
|---|---|---|---|---|
| Women | Self-management | Chronic | Qualitative | Barrier* |
| Woman | | Long-term | | Facilitator* |
| Female | | Disease | | |
| | | Illness | | |
| | | Condition | | |
| | | Health | | |

For peer review only

Eligibility criteria

Utilising the criteria detailed in table two, the reviewer will assess each identified publication's eligibility for inclusion. A PRISMA flow diagram will be used to present the screening process including the number of studies excluded and reasons for this to ensure transparency and reproducibility [15].

**Table Two Eligibility criteria**

| Criteria | Explanation | Justification |
|---|---|---|
| Studies where participants are women | Studies with data from both men and women can be included if the findings are differentiated between men and women's responses. | POP is a condition which solely affects women. Therefore, to ensure the findings of this review can be generalised to the relevant population, it is necessary to limit included studies in case there are differences between the barriers and facilitators men and women report and experience to self-management interventions. |
| Studies where participants are aged 18 or over | Studies with data from individuals over and under 18 years can be included if the findings are differentiated between age groups. | The incidence of POP amongst women aged under 18 years is extremely low [16]. Therefore, the focus of research exploring pessary self-management will be women aged over 18 years. To ensure the findings of this review can be generalised to the relevant population, it is necessary to limit included studies in case there are differences between the barriers and |

For peer review only

| | | |
|---|---|---|
| | | facilitators adult women and young women and girls report and experience to self-management interventions. |
| Studies exploring a self-management intervention | An intervention designed with the aim of improving health, quality of life or perceived self-efficacy for individuals with a specific condition through goal setting, information utilisation, decision making, action and self-reaction [10]. | The terms self-care and self-management have been poorly defined and used interchangeably [9] Therefore, it is necessary to ensure that self-management interventions included within the review meet Creer's definition [10] that a self-management intervention aims to improve health, quality of life or perceived self-efficacy for an individual with a specific health condition through either goal setting, information provision, supported decision making or self-reaction, to ensure the findings are valid. |
| Studies exploring a self-management intervention for a chronic condition | The term chronic disease has been poorly defined within both guidelines and literature leading to confusion [17]. Therefore, to ensure a standardised approach for the purpose of this review, a chronic (also known as a long-term) condition will be defined as a non-communicable condition that lasts for six months or longer and impacts upon quality of life [17-19]. | POP meets this definition of a chronic condition, therefore to ensure the findings of other studies included within the review are likely to be transferable to women with POP, it is necessary to exclude interventions for acute or terminal conditions as the barriers and facilitators cited may differ significantly to individuals with chronic conditions. |

For peer review only

| | | |
|---|---|---|
| Studies which are qualitative research | Original qualitative research. Qualitative studies, mixed methods studies and data collected by questionnaire can be included if answered in free text and analysed using a qualitative approach. | Qualitative research is the most appropriate evidence to explore the barriers and facilitators to self-management as it offers insight into the experiences and perceptions of individuals [13]. |
| Studies which have findings related to the barriers or facilitators to a self-management intervention | The results will include findings about the barriers and facilitators which influence uptake and/or maintenance and /or concordance with a self-management intervention. | The aim of the review is to identify the barriers and facilitators to self-management interventions. |
| Studies with finding directly reported by the target population of women | Data about barriers and facilitators to a self-management intervention must be collected directly from the study population rather than healthcare professionals, partners, family members or carers. | This systematic review aims to identify barriers and facilitators to self-management interventions from the perspective of the women they are designed for. Therefore, the perspectives of healthcare professionals, partners, family members or carers will not be included as these interpretations may not accurately reflect the experiences of women. |

For peer review only

| | | |
|---|---|---|
| Language | Studies must be published in the English language | For pragmatic reasons, studies published in languages other than English will be excluded from the review due to the cost and time required for translation. |
| Original research | Publications that are not original research including reviews, case reports and commentary articles will not be included. | Non-original research will be excluded to avoid duplication of results and ensure all included publications provide rigorous evidence rather than subjective opinion. |
| Date range | Eligible studies will be published between 2005 and the date of the search. | Since 2005, United Kingdom (UK) key health policy demonstrates a distinct change from a traditional medical model approach to healthcare to a person-centred approach where individuals are empowered to self-care and self-manage their long-term conditions with the goal of providing a more efficient service [12, 20, 21]. The NHS Long-term plan, launched in 2019, refers to supported self-management as a means to meet healthcare delivery goals and improve patient care [22], demonstrating that self-management remains a key aspect of national healthcare policy. Therefore, the time parameters for the search will be from 2005 until the search date. It is anticipated this will ensure review findings are sufficiently current to reflect recent practice and experience of women. |

For peer review only

Data management and selection

Following the search, all identified abstracts will be uploaded to reference manager software, following which duplicates and non-original research publications will be removed. The abstracts will then be reviewed for relevance to the review question and in accordance with the eligibility criteria. A sample of 20% of abstracts will be screened by an independent reviewer to ensure concordance with decisions about eligibility. In the instance of disagreement regarding included or excluded studies not resolved through discussion, a third reviewer will be asked to make the final decision. Potentially relevant sources will be retrieved in full and assessed in detail against the inclusion criteria. Reasons for exclusion of sources of evidence that do not meet the inclusion criteria will be recorded and reported in the systematic review. The reference list of all review articles identified in the search and excluded due to not being original research, will be checked and all relevant original research cited will be reviewed for potential inclusion within this review.

Data collection

Data will be extracted using a data extraction tool developed by the reviewers based upon qualitative systematic review guidance [14, 23]. The extracted data will be entered into the tool electronically via Microsoft Excel. The data extraction form will include details of the author(s), year of publication, methodology utilised, theoretical orientation of the researchers, population studied, the self-management intervention, the chronic condition targeted, and key findings relevant to the barriers and facilitators of the self-management intervention. Study findings will be recorded in two separate columns to differentiate between first and second order constructs maintaining clarity about what findings are raw data and which are researcher interpretations [14].

A second reviewer will perform data extraction from a subset of 10% of included articles to ensure a standardised, reproducible approach. In the instance of discrepancies in data extraction processes, these will be explored, discussed and changes made to the data extraction tool if necessary. Any amendments to the data extraction tool will be recorded and published with the review findings to ensure transparency. The final version of the data extraction tool including extracted data will be published alongside the review findings to ensure reproducibility.

For peer review only

Data synthesis

The extracted data will be analysed using a thematic approach. Findings will be coded line by line to identify emerging and recurring descriptive themes amongst the studies [24]. Once all extracted data has been coded, the descriptive themes identified from the coding will be further analysed for broader analytic themes that span the included studies [24]. A number of qualitative systematic reviews of barriers and facilitators to healthy behaviour have used thematic synthesis [25]. Therefore, the synthesis will be performed utilising this established process to ensure an established inductive and deductive approach [25].

Quality appraisal

Qualitative findings offers insight into individuals' views, experiences and perspectives and therefore do not attempt to be generalisable or replicable [26]. However, rigour can achieved in qualitative research by ensuring findings are trustworthy, authentic, typical, transferable and valid [26]. Butler *et al* [14] describe their use of the Critical Appraisal Skills Programme (CASP) qualitative checklist [27]. CASP specify they have not suggested a scoring system for appraising qualitative studies as this was not the purpose of the checklist [27]. However, Butler *et al* [14] explain the scoring system they successfully used for each checklist criterion and overall scoring to determine the level of quality of each individual study. Therefore, this same scoring system of the CASP checklist will be utilised (Table Three). As this review aims to provide an overview of the current evidence base, all studies will be included regardless of the appraised quality [14]. However, as advocated by Long, French [28] a hierarchical approach to thematic analysis will be undertaken with the quality of a study informing whether or not new codes are created. For this review, new codes will be created for high and medium quality studies. However, for low and very low-quality studies, no new codes will be created but findings utilised to support themes identified from codes created through thematic analysis of higher quality studies. This will ensure finding are weighted appropriately based upon the methodological quality of the studies [28]. Furthermore, an overview of strengths and limitations of the identified evidence base will be provided to highlight any patterns in quality, or lack of it, and inform the methodological design of future research within this subject area.

For peer review only

**Table Three-Quality Appraisal Scoring System**

| CASP Scoring system | |
|---|---|
| **Checklist criterion** | |
| Yes | 1 |
| No | 0 |
| Unsure | 0.5 |
| **Overall score** | |
| High quality | 9-10 |
| Moderate quality | 7.5-8.5 |
| Low quality | 6- ≤7.5 |
| Very low quality | ≤6 |

Another issue with the synthesis of findings from qualitative studies is that the subjectivity of study findings means our confidence in cumulative evidence synthesised from included studies is reliant upon decisions and interpretations made by authors. While quality appraisal using the CASP tool assesses research processes, it does not determine the credibility or dependability of interpretations made by the researcher which may be influenced by personal bias [29]. Because of this, researchers from the Joanna Briggs Institute developed a system called ConQual which enables assessment and scoring of a study's credibility, dependability and therefore overall confidence in the synthesised findings [29]. Using the ConQual, statements made from the findings of synthesised data will be presented in tabular format alongside a ConQual rating of credibility, dependability and overall score to ensure conclusions made can be weighted accordingly.

Outcomes and prioritisation

The experiences and perceptions of women reporting barriers and facilitators to self-management interventions targeting chronic diseases are the focus of this review. Therefore, all, barriers or facilitators to the uptake or maintenance of a self-management intervention for a chronic condition reported within identified and eligible studies will be included within data synthesis.

For peer review only

Data Analysis and Presentation

Details of the studies identified by the search, for example the extent of identified literature, the context of included research such as the methodology utilised, philosophical standpoint, country of origin, study population, self-management intervention and chronic disease targeted, will be presented in numerical and tabular format to provide an overview of the evidence base. A CASP and ConQual ratings will also be presented alongside each included study to enable readers to take in to account the quality, dependability, credibility and overall confidence in each study and therefore synthesised findings. Themes identified will be described in both tabular and text format to ensure transparency and clarity in the process of thematic analysis and identified themes, in addition to more detailed discussion and demonstration of evidence supporting identified themes within the text.

Confidence in cumulative evidence

One criticism of the synthesis of qualitative studies is that the nature of qualitative research means the context of each study may vary significantly, impacting upon study findings which creates a potential for bias when performing meta-synthesis [24]. To minimise this, the extraction tool will include details of each study's context and be presented alongside the review findings, enabling readers to contextualise study findings as advocated by Thomas and Harden [24]. Further sub-analysis will be undertaken to explore whether certain contexts, for example the age of a study population, type of self-management intervention or chronic condition correlate with specific themes. This will further understanding about whether identified barriers or facilitators relate to a specific context or have broader applicability [24].

Patient and public involvement

Members of the public have not directly been involved with development of this protocol or review process. However, the need for research exploring pessary self-management was highlighted by The James Lind Alliance (JLA) Priority Setting Partnership for pessary and prolapse [30]. Several women with experience of pessaries participated in this partnership either as members of the steering group, by attending the consensus workshop or completing questionnaires. Understanding more about pessary self-management was

For peer review only

ranked third out of 20 priorities by the JLA Priority Setting Partnership. This systematic review will inform a research project exploring pessary self-management which has been identified and prioritised by patients and members of the public.

Ethics and dissemination

No ethical or Health Research Authority (HRA) approval is required to undertake the systematic review. The systematic review findings will be disseminated by publication in a peer reviewed journal. The findings will also inform subsequent exploratory work regarding pessary self-management.

For peer review only

Authors contributions

Lucy Dwyer devised the systematic review question, methodology and drafted this manuscript.

Dawn Dowding substantively contributed to the development of the systematic review question, methodology and revised and approved this manuscript.

Rohna Kearney substantively contributed to the development of the systematic review question, methodology and revised and approved this manuscript.

Competing interests statement

Lucy Dwyer and Rohna Kearney are co-applicants of the NIHR/HTA funded Treatment of Prolapse with Self-Care Pessary (TOPSY) study.

Funding

Lucy Dwyer, Clinical Doctoral Research Fellow, NIHR300519 is funded by Health Education England (HEE) / National Institute for Health Research (NIHR) for this research project. The views expressed in this publication are those of the author(s) and not necessarily those of the NIHR, NHS or the UK Department of Health and Social Care.

For peer review only

## References


1. Haylen, B.T., et al., *An International Urogynecological Association (IUGA) / International Continence Society (ICS) Joint Report on the Terminology for Female Pelvic Organ Prolapse (POP)*, in *Neurourology and Urodynamics*. 2016.
2. Bugge, C., et al., *Pessaries (mechanical devices) for managing pelvic organ prolapse in women.* Cochrane Database Syst Rev, 2020. **11**(11): p. Cd004010.
3. Lone, F., R. Thakar, and A.H. Sultan, *One-year prospective comparison of vaginal pessaries and surgery for pelvic organ prolapse using the validated ICIQ-VS and ICIQ-UI (SF) questionnaires.* International Urogynecology Journal, 2015. **26**(9).
4. Miceli, A. and J.L. Dueñas-Diez, *Effectiveness of ring pessaries versus vaginal hysterectomy for advanced pelvic organ prolapse. A cohort study.* International Urogynecology Journal, 2019. **30**(12).
5. Kearney, R. and C. Brown, *Self-management of vaginal pessaries for pelvic organ prolapse.* BMJ Quality Improvement Reports, 2014. **3**(1).
6. Basu, M., B. Wise, and J. Duckett, *A qualitative study of women's preferences for treatment of pelvic floor disorders.* BJOG: An International Journal of Obstetrics & Gynaecology, 2011. **118**(3): p. 338-344.
7. Nguyen, J.N. and C.R. Jones, *Pessary treatment of pelvic relaxation: Factors affecting successful fitting and continued use.* Journal of Wound, Ostomy and Continence Nursing, 2005. **32**(4).
8. Bugge, C., et al., *Does self-management of vaginal pessaries improve care for women with pelvic organ prolapse?*, in *The BMJ*. 2021.
9. Rijken, M., et al., *Supporting self-management.* Caring for people with chronic conditions: a health system perspective, 2008: p. 116-142.
10. Creer, T.L., *Self-management of chronic illness*, in *Handbook of self-regulation*. 2000, Elsevier. p. 601-629.
11. Organization, W.H., *Health education in self-care: Possibilities and limitations*. 1984, World Health Organization.
12. Department of Health, *Self Care: A Real Choice*, London: Department of Health Publications, Editor. 2005.
13. Gray, J. and S. Grove, *Burns and Grove's the practice of nursing research : appraisal, synthesis, and generation of evidence*. 2021.
14. Butler, A., H. Hall, and B. Copnell, *A Guide to Writing a Qualitative Systematic Review Protocol to Enhance Evidence-Based Practice in Nursing and Health Care.* Worldviews Evid Based Nurs, 2016. **13**(3): p. 241-9.
15. Moher, D., et al., *Preferred reporting items for systematic review and meta-analysis protocols (PRISMA-P) 2015 statement.* Systematic Reviews, 2015. **4**(1): p. 1.
16. Hines, M., R. High, and J.M. Danford, *Management of Pelvic Floor Disorders in Young Women.* Current Bladder Dysfunction Reports, 2020. **15**(4): p. 259-266.
17. Bernell, S. and S.W. Howard, *Use Your Words Carefully: What Is a Chronic Disease?* Frontiers in public health, 2016. **4**: p. 159-159.
18. Department of Health, *Long Term Conditions Compendium of Information*. 2012.
19. O'Halloran, J., G.C. Miller, and H. Britt, *Defining chronic conditions for primary care with ICPC-2.* Family Practice, 2004. **21**(4): p. 381-386.
20. Department of Health, *Supporting people with long term conditions to self care – A guide to developing local strategies and good practice*, T.D.o. Health, Editor. 2006.
21. Department of Health, *The NHS Plan: a plan for investment a plan for reform*, D.o. Health, Editor. 2007.
22. Nhs, *The NHS Long Term Plan*. 2019.
23. Timulak, L., *Meta-analysis of qualitative studies: A tool for reviewing qualitative research findings in psychotherapy.* Psychotherapy Research, 2009. **19**(4-5): p. 591-600.


For peer review only

24. Thomas, J. and A. Harden, *Methods for the thematic synthesis of qualitative research in systematic reviews.* BMC Medical Research Methodology, 2008. **8**(1): p. 45.
25. Kanavaki, A.M., et al., *Barriers and facilitators to physical activity in people with hip or knee osteoarthritis: protocol for a systematic review of qualitative evidence.* BMJ Open, 2016. **6**(11): p. e012049.
26. Holloway, I. and K. Galvin, *Qualitative research in nursing and healthcare*. 2016: John Wiley & Sons.
27. Critical Appraisal Skills Programme (CASP). *Qualitative checklist*. 2018 08/01/22]; Available from: https://casp-uk.net/wp-content/uploads/2018/01/CASP-Qualitative-Checklist-2018.pdf.
28. Long, H.A., D.P. French, and J.M. Brooks, *Optimising the value of the critical appraisal skills programme (CASP) tool for quality appraisal in qualitative evidence synthesis.* Research Methods in Medicine & Health Sciences, 2020. **1**(1): p. 31-42.
29. Munn, Z., et al., *Establishing confidence in the output of qualitative research synthesis: the ConQual approach.* BMC Medical Research Methodology, 2014. **14**(1): p. 108.
30. Lough, K., et al., *Shared research priorities for pessary use in women with prolapse: Results from a James Lind Alliance Priority Setting Partnership.* BMJ Open, 2018. **8**(4).

For peer review only

## Supplementary material One

# PRISMA 2020 Checklist

| Section and Topic | Item # | Checklist item | Location where item is reported |
|---|---|---|---|
| **TITLE** | | | |
| Title | 1 | Identify the report as a systematic review. | Page 1 |
| **ABSTRACT** | | | |
| Abstract | 2 | See the PRISMA 2020 for Abstracts checklist. | Page 2 |
| **INTRODUCTION** | | | |
| Rationale | 3 | Describe the rationale for the review in the context of existing knowledge. | Page 4 |
| Objectives | 4 | Provide an explicit statement of the objective(s) or question(s) the review addresses. | Page 5 |
| **METHODS** | | | |
| Eligibility criteria | 5 | Specify the inclusion and exclusion criteria for the review and how studies were grouped for the syntheses. | Page 8 |
| Information sources | 6 | Specify all databases, registers, websites, organisations, reference lists and other sources searched or consulted to identify studies. Specify the date when each source was last searched or consulted. | Page 7 |
| Search strategy | 7 | Present the full search strategies for all databases, registers and websites, including any filters and limits used. | To be included with results |
| Selection process | 8 | Specify the methods used to decide whether a study met the inclusion criteria of the review, including how many reviewers screened each record and each report retrieved, whether they worked independently, and if applicable, details of automation tools used in the process. | Page 12 |
| Data collection process | 9 | Specify the methods used to collect data from reports, including how many reviewers collected data from each report, whether they worked independently, any processes for obtaining or confirming data from study investigators, and if applicable, details of automation tools used in the process. | Page 12 |
| Data items | 10a | List and define all outcomes for which data were sought. Specify whether all results that were compatible with each outcome domain in each study were sought (e.g. for all measures, time points, analyses), and if not, the methods used to decide which results to collect. | Page 12 |
| | 10b | List and define all other variables for which data were sought (e.g. participant and intervention characteristics, funding sources). Describe any assumptions made about any missing or unclear information. | Page 12 |
| Study risk of bias assessment | 11 | Specify the methods used to assess risk of bias in the included studies, including details of the tool(s) used, how many reviewers assessed each study and whether they worked independently, and if applicable, details of automation tools used in the process. | Page 13-14 |
| Effect measures | 12 | Specify for each outcome the effect measure(s) (e.g. risk ratio, mean difference) used in the synthesis or presentation of results. | Page 13-14 |
| Synthesis methods | 13a | Describe the processes used to decide which studies were eligible for each synthesis (e.g. tabulating the study intervention characteristics and comparing against the planned groups for each synthesis (item #5)). | Page 15 |
| | 13b | Describe any methods required to prepare the data for presentation or synthesis, such as handling of missing summary statistics, or data conversions. | Page 15 |
| | 13c | Describe any methods used to tabulate or visually display results of individual studies and s 4yntheses. | Page 15 |
| | 13d | Describe any methods used to synthesize results and provide a rationale for the choice(s). If meta-analysis was performed, describe the model(s), method(s) to identify the presence and extent of statistical heterogeneity, and software package(s) used. | Page 14-15 |

For peer review only - http://bmjopen.bmj.com/site/about/guidelines.xhtml

## Supplementary material One

# PRISMA 2020 Checklist

| Section and Topic | Item # | Checklist item | Location where item is reported |
|---|---|---|---|
| | 13e | Describe any methods used to explore possible causes of heterogeneity among study results (e.g. subgroup analysis, meta-regression). | Page 15 |
| | 13f | Describe any sensitivity analyses conducted to assess robustness of the synthesized results. | Page 15 |
| Reporting bias assessment | 14 | Describe any methods used to assess risk of bias due to missing results in a synthesis (arising from reporting biases). | Page 15 |
| Certainty assessment | 15 | Describe any methods used to assess certainty (or confidence) in the body of evidence for an outcome. | Page 15 |
| **RESULTS** | | | |
| Study selection | 16a | Describe the results of the search and selection process, from the number of records identified in the search to the number of studies included in the review, ideally using a flow diagram. | To be included with results |
| | 16b | Cite studies that might appear to meet the inclusion criteria, but which were excluded, and explain why they were excluded. | To be included with results |
| Study characteristics | 17 | Cite each included study and present its characteristics. | To be included with results |
| Risk of bias in studies | 18 | Present assessments of risk of bias for each included study. | To be included with results |
| Results of individual studies | 19 | For all outcomes, present, for each study: (a) summary statistics for each group (where appropriate) and (b) an effect estimate and its precision (e.g. confidence/credible interval), ideally using structured tables or plots. | To be included with results |
| Results of syntheses | 20a | For each synthesis, briefly summarise the characteristics and risk of bias among contributing studies. | To be included with results |
| | 20b | Present results of all statistical syntheses conducted. If meta-analysis was done, present for each the summary estimate and its precision (e.g. confidence/credible interval) and measures of statistical heterogeneity. If comparing groups, describe the direction of the effect. | To be included with results |
| | 20c | Present results of all investigations of possible causes of heterogeneity among study results. | To be included with results |
| | 20d | Present results of all sensitivity analyses conducted to assess the robustness of the synthesized results. | To be included with results |

For peer review only

## Supplementary material One

# PRISMA 2020 Checklist

| Section and Topic | Item # | Checklist item | Location where item is reported |
|---|---|---|---|
| Reporting biases | 21 | Present assessments of risk of bias due to missing results (arising from reporting biases) for each synthesis assessed. | To be included with results |
| Certainty of evidence | 22 | Present assessments of certainty (or confidence) in the body of evidence for each outcome assessed. | To be included with results |
| **DISCUSSION** | | | |
| Discussion | 23a | Provide a general interpretation of the results in the context of other evidence. | To be included with results |
| | 23b | Discuss any limitations of the evidence included in the review. | To be included with results |
| | 23c | Discuss any limitations of the review processes used. | To be included with results |
| | 23d | Discuss implications of the results for practice, policy, and future research. | To be included with results |
| **OTHER INFORMATION** | | | |
| Registration and protocol | 24a | Provide registration information for the review, including register name and registration number, or state that the review was not registered. | Page 6 |
| | 24b | Indicate where the review protocol can be accessed, or state that a protocol was not prepared. | This publication |
| | 24c | Describe and explain any amendments to information provided at registration or in the protocol. | n/a |
| Support | 25 | Describe sources of financial or non-financial support for the review, and the role of the funders or sponsors in the review. | Page 17 |
| Competing interests | 26 | Declare any competing interests of review authors. | Page 17 |
| Availability of data, code and other materials | 27 | Report which of the following are publicly available and where they can be found: template data collection forms; data extracted from included studies; data used for all analyses; analytic code; any other materials used in the review. | To be included with results |

*From:* Page MJ, McKenzie JE, Bossuyt PM, Boutron I, Hoffmann TC, Mulrow CD, et al. The PRISMA 2020 statement: an updated guideline for reporting systematic reviews. BMJ 2021;372:n71. doi: 10.1136/bmj.n71

For more information, visit: http://www.prisma-statement.org/

For peer review only

Supplementary material Two

Systematic Review Search Strategy

1. women.mp. or Women/
2. woman.mp. or Women/
3. Female/
4. 1 or 2 or 3
5. self-management.mp. or Self-Management/
6. 4 and 5
7. qualitative.mp.
8. 6 and 7
9. chronic.mp.
10. long-term.mp.
11. condition.mp.
12. illness.mp.
13. condition.mp.
14. Health/ or health.mp.
15. 9 or 10 or 11 or 12 or 13 or 14
16. 8 and 15
17. barrier*.mp.
18. facilitator*.mp.
19. 17 or 18
20. 16 and 19
21. limit 20 to english language
22. limit 21 to yr="2005 -Current"

For peer review only
